# Akt phosphorylates Prohibitin 1 to mediate its mitochondrial localization and promote proliferation of bladder cancer cells

**DOI:** 10.1038/cddis.2015.40

**Published:** 2015-02-26

**Authors:** L Jiang, P Dong, Z Zhang, C Li, Y Li, Y Liao, X Li, Z Wu, S Guo, S Mai, D Xie, Z Liu, F Zhou

**Affiliations:** 1 State Key Laboratory of Oncology in South China, Sun Yat-sen University Cancer Center, Collaborative Innovation Center for Cancer Medicine, Guangdong 510060, China; 2 Department of Urology, Sun Yat-sen University Cancer Center, Guangzhou, Guangdong 510060, China; 3 Department of Pathology, Sun Yat-sen University Cancer Center, Guangzhou, Guangdong 510060, China

## Abstract

Bladder cancer (BC) is very common and associated with significant morbidity and mortality, though the molecular underpinnings of its origination and progression remain poorly understood. In this study, we demonstrate that Prohibitin 1 (PHB) was overexpressed in human BC tissues and that PHB upregulation was associated with poor prognosis. We also found that PHB was necessary and sufficient for BC cell proliferation. Interestingly, the overexpressed PHB was primarily found within mitochondria, and we provide the first direct evidence that phosphorylation by Akt at Thr258 of PHB induces this mitochondrial localization. Inhibiton of Akt reverses these effects and inhibited the proliferation of BC cells. Finally, the phosphorylation of PHB was required for BC cell proliferation, further implicating the importance of the Akt in BC. Taken together, these findings identify the Akt/PHB signaling cascade as a novel mechanism of cancer cell proliferation and provide the scientific basis for the establishment of PHB as a new prognostic marker and treatment target for BC.

Bladder cancer (BC) is the one of the most common malignancies of the urinary tract system and represents a significant cause of morbidity and mortality.^1^ Many studies have shown that the progression of BC is highly associated with metastasis,^2, 3, 4^ which involves tumorigenesis, migration and invasion. Unlike other urological cancers, BC lacks clinically useful biomarkers for predicting disease and clinical outcome.^5, 6^

Prohibitin 1 (PHB) has been shown to significantly impact cellular senescence and development, as well as suppression of tumor cell proliferation.^7, 8, 9, 10, 11^ Unfortunately, loss of prohibitin subunits is associated with embryonic lethality in multicellular organisms such as *C. elegans*^12^ and mice,^13, 14^ hampering functional studies on mammalian prohibitins at the organismal level. Knockdown experiments at the cellular level, however, have revealed that PHB may be essential for cell proliferation.^14, 15^ These findings are in striking contrast to the previously proposed antiproliferative role of PHB and the predicted function as a negative regulator of E2F-mediated transcription,^10, 11^ though the difference in localization of PHB may explain the discrepancy. PHB has been visualized in the mitochondria,^16, 17^ nucleus^11, 18, 19^ and plasma membrane^20, 21^ in different mammalian cell lines, although the exact role of PHB in each location remains unclear.

RAC-alpha serine/threonine-protein kinase (Akt) is responsible for mediating a variety of biological responses.^22, 23, 24^ Dysregulation of Akt activity has been implicated in the pathogenesis of a growing number of disorders.^24^ Recently, PHB has been identified as a substrate for Akt in MiaPaCa-2 clones and HEK-293T cells expressing constitutively active Akt.^25^ In these cells, Akt-induced phosphorylation of PHB occured at Thr 258. This phosphorylation of PHB by Akt could lead to alteration in the localization of PHB, transcriptional regulation of E2F and also activation of the Ras-Raf pathway.^26^ Considering these possibilities, it would be both interesting and clinically relevant to study the potential phosphorylation of PHB by endogenous Akt.

The purpose of this study was to investigate the role of Akt in PHB-induced cell proliferation in human BC tissue and cultured cells. This study is the first to show that endogenous Akt has the ability to phosphorylate PHB, thereby inducing BC cell proliferation, introducing a new molecular mechanism to study and target in BC.

## Results

### PHB upregulation is associated with poor prognosis in BC patients

**Figure 1:**
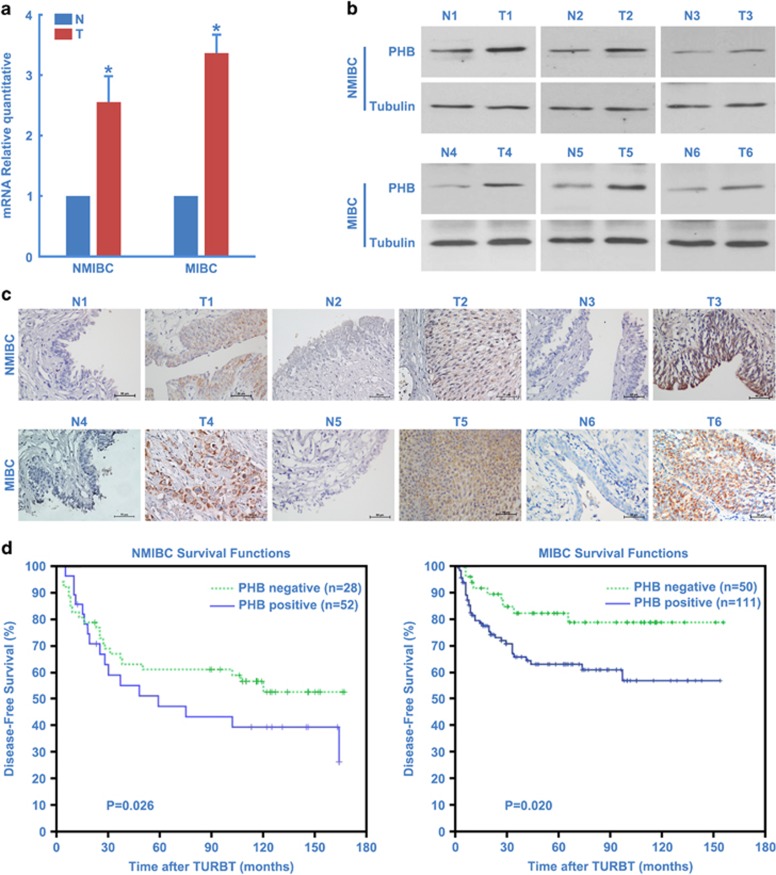
PHB upregulation is associated with poor prognosis in BC patients. (**a**) Average matched tumor-adjacent morphologically normal bladder epithelial tissue/tumor tissue (N/T) ratios of PHB expression were quantified by Q-PCR and normalized against GAPDH (glyceraldehyde 3-phosphate dehydrogenase). (**b**) Western blotting analysis of PHB expression in each of the matched adjacent normal bladder epithelial (N) and BC tissue (T). (**c**) IHC confirmed that PHB protein was upregulated in the BC tissue (T) compared with the paired adjacent normal bladder epithelium (N) from the same patient. Scale bar, 50 *μ*m. (**d**) Kaplan–Meier curves with univariate analyses (log-rank) for (**d**) NMIBC and MIBC patients. Patients with positive PHB expression had a cumulative 5 years disease-free survival rate of 63.0% (HR: 2.307, 95% confidence interval, 1.114–4.780), compared with 82.1% for patients with negative PHB expression on MIBC patients. Error bars represent the S.D calculated from three parallel experiments. *Denotes *P*<0.05

In a previous study, overexpression of PHB was observed in >80% of BC cases, supporting its relevance in the pathogenesis of BC.^27^ We confirmed that PHB was markedly upregulated at both the transcriptional and translational levels in BC tissues compared with adjacent normal urothelial tissues (Figures 1a and b). Importantly, analysis of the normal/tumor (N/T) ratio revealed that N/T of PHB mRNA and protein were significantly higher in muscle invasive bladder cancer (MIBC) tissues compared with nonmuscle invasive bladder cancer (NMIBC) tissues. These results were further confirmed by immunohistochemical analysis, which also showed apparent upregulation of PHB in 241 BC tissue specimens (Figures 1c and d).

Correlation analysis demonstrated that the overexpression of PHB was positively correlated with a significantly lower disease-free survival rate than with negative expression of PHB in the NMIBC cohort (Figure 1d). There was also significant association between PHB expression and advanced pT and pN classification in MIBC (Table 1). Kaplan–Meier analysis showed that the mean disease-free time for patients with MIBC and overexpression of PHB was 110 months compared with 142 months for patients with normal expression of PHB (*P*=0.020, log-rank test, Figure 1d). Further multivariate Cox regression analysis determined that PHB expression is an independent prognostic factor for the poor survival of MIBC patients (HR 1.176, CI 0.700–1.973, *P*=0.045, Table 2). In conclusion, survival analysis shows a significant correlation between increased PHB expression and poor prognosis in BC patients.

### PHB is necessary and sufficient for BC cell proliferation

PHB is an evolutionarily conserved and ubiquitous protein that may be involved in tumorigenesis and development of BC.^27, 28^ Based on the obvious upregulation of PHB in BC tissues, we hypothesized that the high expression of PHB in BC cells is necessary for cancer cell proliferation.

**Figure 2:**
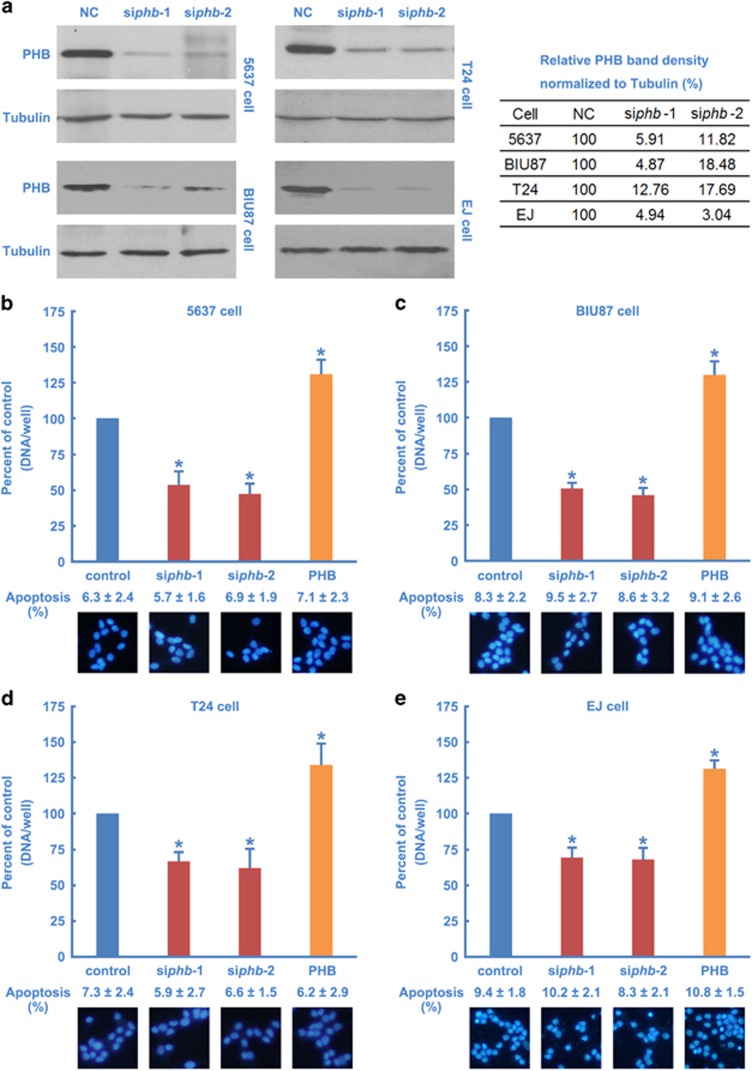
PHB is necessary and sufficient for BC cell proliferation. (**a**) The efficacy and specificity of PHB siRNAs. 5637, BIU87, T24 or EJ cells were transfected with PHB siRNAs or negative control siRNAs for 24 h. The cell lysates were harvested and subjected to western blotting using the indicated antibodies (left), and then the results were quantified (right). (**b**–**e**, upper panel) 5637, BIU87, T24 and EJ cells were transfected with PHB siRNAs or negative control siRNAs for 24 h. Silencing endogenous PHB inhibited cell growth, and cell proliferation was quantified. (**b**–**e**, upper panel) Overexpression PHB in 5637, BIU87, T24 or EJ cells for 24 h, and then cell proliferation was quantified. (**b**–**e**, lower panel) 5637, BIU87, T24 or EJ cells were treated as in the upper panel of panels **b**–**e**, then stained with Hoechst 33258 and cell apoptosis rate was quantified. The values represent the mean±S.E. of at least three independent experiments. *Denotes *P*<0.05

To determine the role of PHB in BC cell proliferation, four BC (5637, BIU87, T24 and EJ) cell lines were selected for investigation *in vitro*. Two siRNAs were designed to reduce the levels of BC cell PHB protein (Figure 2a). A remarkable reduction in cell proliferation was observed in cells after the siRNA knockdown of PHB (Figures 2b–e). Gene silencing of PHB did not promote BC cell apoptosis (Figures 2b–e). Interestingly, Theiss and colleagues have reported that gene silencing of PHB in epithelial cells mainly induces autophagy,^29^ confirming that knockdown of PHB would not induce remarkable cell apoptosis. These results are consistent with previous studies^17^ and confirm that PHB is required for BC cell proliferation.

To determine whether PHB is sufficient to upregulate BC cell proliferation, the effect of wild-type PHB on the proliferation of cultured cancer cells was examined. Transfection of BC cells with wild-type PHB significantly increased cell proliferation (Figures 2b–e), suggesting that PHB is both necessary and sufficient for BC cell proliferation.

### Upregulated PHB is mainly localized to the mitochondria

Prohibitin has been shown to be localized to different cellular compartments.^14, 18, 20^ The most recent functional studies, however, emphasize the role of mitochondrial-based prohibitins for cellular homeostasis.^17^ The precise molecular function of the PHB complex is not clear, but a role as chaperone for respiration chain proteins or as a general structuring scaffold required for optimal mitochondrial morphology and function are suspected.^17^ Recently, prohibitins have been demonstrated to be positive, rather than negative, regulators of cell proliferation in both plants^30^ and mice.^13, 14^

**Figure 3:**
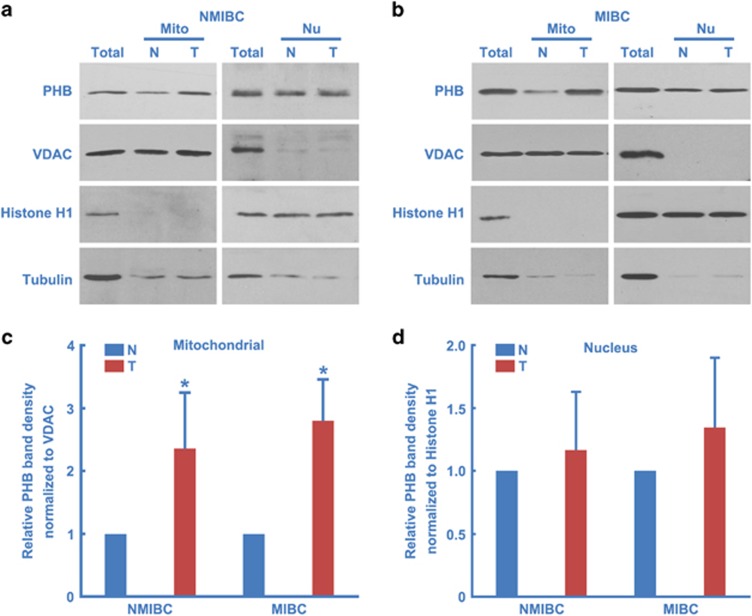
Upregulated PHB was mainly localized in mitochondria. (**a** and **b**) NMIBC or MIBC and matched tumor-adjacent morphologically normal bladder epithelial tissue mitochondria (Mito) and nuclei (Nu) were separated and analyzed by western blotting using PHB, Histone H1 (nucleus marker), VDAC (mitochondrial marker) and Tubulin (cytoplasm marker) antibodies; Total, total tissue lysates. (**c** and **d**) Twenty pairs of PHB-upregulated BC tissues and matched tumor-adjacent morphologically normal bladder epithelial tissue mitochondria and nuclei were separated and analyzed by western blotting as in panels **a** and **b**, and then the results were quantified. N, adjacent normal bladder epithelial; T, BC tissue. The values represent the mean±S.E. of at least three independent experiments. *Denotes *P*<0.05

While delineating how PHB promotes the proliferation of BC cells, we hypothesized that it is through mitochondrial localization that PHB mediates its effects. In normal bladder tissue, the majority of PHB was localized to the nucleus (Figure 3a). In the NMIBC or MIBC tissues, PHB remained in the nucleus (Figures 3a, b and d); however, mitochondrial localization was dramatically increased (Figures 3a–c). To confirm this result, 20 pairs of PHB-upregulated BC tissues and matched tumor-adjacent morphologically normal bladder epithelial tissue mitochondria and nuclei were separated and analyzed by western blotting. In the mitochondria extraction, PHB was upregulated in 17 of the 20 (17/20, 85%) NMIBC tissue samples, upregulated in 19 of the 20 (19/20, 95%) MIBC tissue samples (the normal controls are PHB expression level of tumor-adjacent morphologically normal bladder epithelial tissue mitochondria); in the nuclear extraction, PHB was upregulated in 3 of the 20 (3/20, 15%) NMIBC tissue samples, upregulated in 4 of the 20 (4/20, 20%) MIBC tissue samples (the normal controls are PHB expression level of tumor-adjacent morphologically normal bladder epithelial tissue nuclei). From these data, we conclude that the upregulation of PHB that is observed specifically in NMIBC and MIBC tissues is mostly localized to the mitochondria.

### Akt activates and regulates the mitochondrial localization of PHB

The phosphatidylinositol-4-phosphate 3-kinase (PI3K)/Akt signaling pathway is one of the most frequently dysregulated pathways in cancer and is thought to have a major role in bladder carcinogenesis.^31, 32^ Interestingly, previous studies showed that phosphorylation of PHB by Akt attenuates its interaction with PIP3 and enhances insulin signaling,^33^ but its relationship with BC remain unknown.

**Figure 4:**
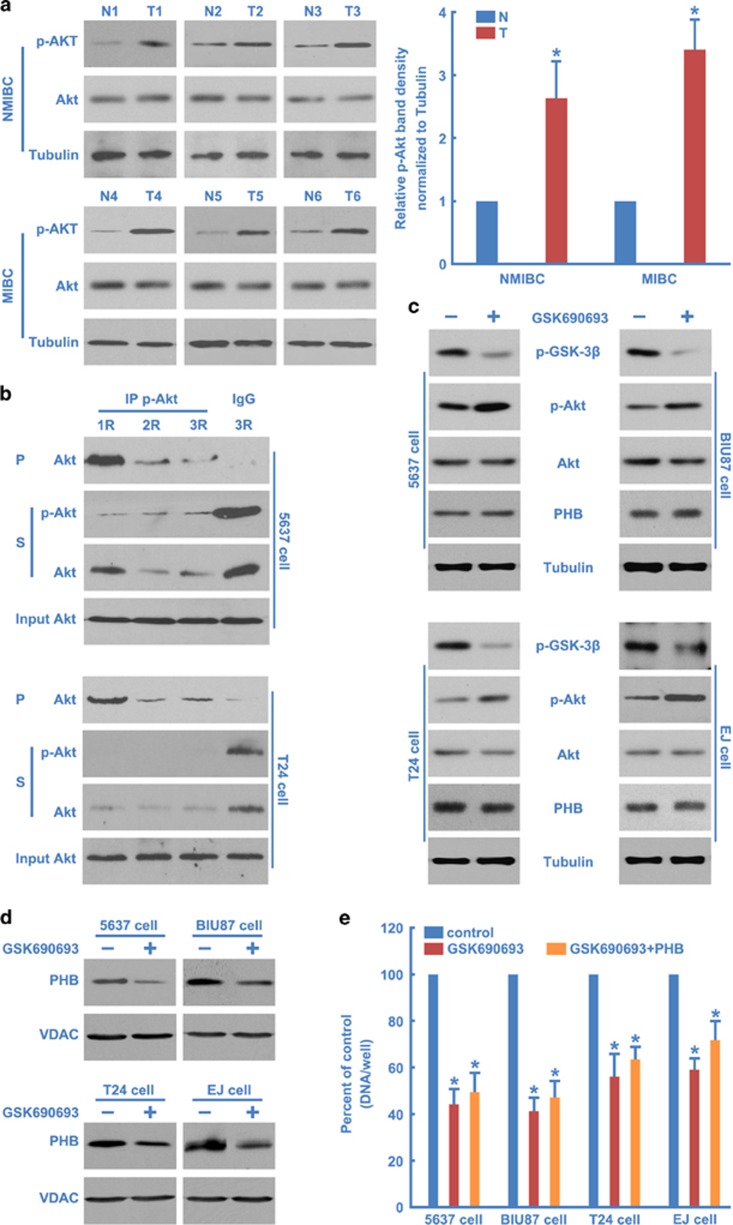
Akt activated and regulates the mitochodnria localization of PHB. (**a**) Western blotting analysis of activated Akt (phospho-Akt Ser473) in each of the matched adjacent normal bladder epithelial (N) and BC tissue (T) (left), and then the results were quantified (right). (**b**) 5637 cells or T24 cells were lysed and subjected to three round immunodepletion analysis using an anti-phospho-Akt Ser473 antibody and analyzed by western blotting using the indicated antibodies. (**c**) 5637, BIU87, T24 or EJ cells were treated with or without Akt inhibitor GSK690693 (5 *μ*M) for 12 h. The cell lysates were harvested and subjected to western blotting using the indicated antibodies. (**d**) 5637, BIU87, T24 or EJ cells were treated with or without Akt inhibitor GSK690693 (5 *μ*M) for 12 h, and then mitochondria (Mito) were separated and analyzed by western blotting using the indicated antibodies. (**e**) 5637, BIU87, T24 or EJ cells were transfected with or without PHB plasmids for 24 h, then were treated with or without Akt inhibitor GSK690693 (5 *μ*M) for 12 h, and cell proliferation was quantified. The values represent the mean±S.E. of at least three independent experiments. *Denotes *P*<0.05

Akt was markedly activated in BC tissues compared with adjacent normal urothelial tissues (Figure 4a). Furthermore, to examine whether Akt is activated in the cultured BC cells, an immunodepletion study was performed. We found that immunodepletion of phosphorylated Akt significantly affected the total Akt in both 5637 and T24 BC cell lines (Figure 4b). Thus Akt appeared to be activated in both BC tissues and cell lines.

To examine whether the activity of Akt is responsible for localization of PHB, BC cell lines were treated with the Akt inhibitor GSK690693, a potent and selective, ATP-competitive, pan-Akt kinase inhibitor currently in clinical development for patients with various malignancies.^34, 35^ Treatment with GSK690693 resulted in reduction in phosphorylation of Akt sustrate GSK3*β* (Ser9) and an increase in AKT phosphorylation at Ser473 site (Figure 4c), consistent with the feedback mechanism observed previously with this AKT kinase inhibitor.^36^ Furthermore, inhibition of Akt activation by GSK690693 effectively reduced the localization of PHB in mitochondria (Figure 4d), without influencing the total protein level of PHB (Figure 4c).

To determine the role of Akt in BC cell proliferation, the Akt inhibitor GSK690693 was used to reduce Akt activity, leading to a significant reduction in cell proliferation after treatment (Figure 4e). Furthermore, we showed that increased cell proliferation during PHB overexpression is eliminated with Akt inhibitor treatment (Figure 4e). These results are consistent with previous studies and confirm that Akt is required for BC cell proliferation.

### Akt mediates the phosphorylation of PHB at Thr258

**Figure 5:**
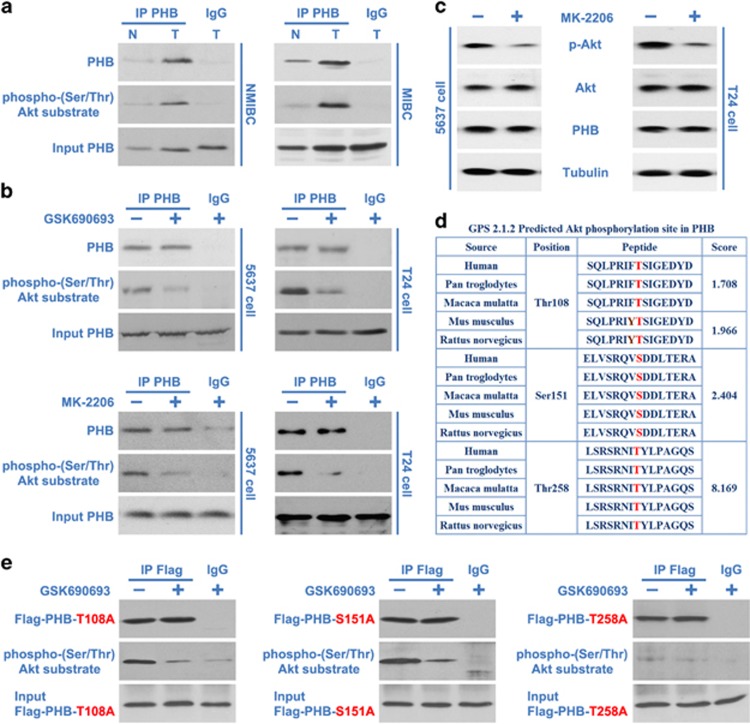
Akt mediates the phosphorylation of PHB at Thr258. (**a**) PHB immunoprecipitates of matched normal bladder or NMIBC or MIBC tissues were probed with Phospho-(Ser/Thr) Akt substrate (PAS) and PHB antibody. (**b**) 5637 or T24 cells were treated with or without Akt inhibitor GSK690693 (5 *μ*M) or MK-2206 (5 *μ*M) for 12 h were lysed and subjected to immunoprecipitation analysis using a PHB antibody, and then PHB immunoprecipitates were probed with Phospho-(Ser/Thr) Akt substrate (PAS) antibody. (**c**) 5637 or T24 cells were treated with or without Akt inhibitor MK-2206 (5 *μ*M) for 12 h. The cell lysates were harvested and subjected to western blotting using the indicated antibodies. (**d**) Sequence analysis revealed that human PHB contains three Akt consensus phosphorylation site at Thr108, Ser151 and Thr258. (**e**) T24 cells were transfected with Flag-PHB T108A, Flag-PHB T151A or Flag-PHB T258A and treated with or without Akt inhibitor GSK690693 (5 *μ*M) for 12 h. The cells were then lysed and Flag-PHB T108A, Flag-PHB T151A or Flag-PHB T258A were immunoprecipitated, normalized to the same protein quantity and subjected to western blotting and probed with Phospho-(Ser/Thr) Akt substrate (PAS) antibody. N, adjacent normal bladder epithelial; T, BC tissue. The results are representative of three independent experiments

Increased cell proliferation during PHB overexpression is eliminated with Akt inhibitor treatment (Figure 4e), which indicates that Akt may promote cell proliferation in a PHB-dependent manner. Previous work and our study have also established that Akt is activated and has a crucial role in the localization of PHB and cell proliferation in BC,^31^ prompting us to ask whether PHB is an Akt substrate in BC cells. Phospho-(Ser/Thr) Akt substrate (PAS) antibody recognizes the RXRXXp(S/T) peptide motif and has been used in studies of Akt substrates. When PHB immunoprecipitates of normal bladder, NMIBC or MIBC tissues were probed with PAS antibody, we showed that the phosphorylation of PHB in NMIBC or MIBC tissues controlled to normal bladder tissues were remarkably upregulated (Figure 5a). Furthermore, PAS antibody also detected phosphorylated PHB in PHB immunoprecipitates of BC cells, while GSK690693 inhibited the phosphorylation of PHB (Figure 5b). The level of PHB phosphorylation correlates well with that of Akt activity in BC tissues and cells. Another Akt inhibitor MK-2206 (MK-2206 is an allosteric inhibitor and is activated by the pleckstrin homology domain and inhibits auto-phosphorylation of both Akt Thr308 and Ser473),^37^ which inhibit Akt activation and did not perturb the expression of PHB (Figure 5c), also inhibited the phosphorylation of PHB effectively (Figure 5b). Our data confirm that PHB is a downstream effector of the PI3K/Akt pathway in BC cells and are consistent with the earlier finding of the breast cancer cell line, MCF-7.

To identify the putative site(s) of PHB phosphorylated by Akt, PHB complete amino-acid sequences were scanned by the Scansite databases (using phosphorylation sites identification software: Group-based Prediction System (GPS), ver 2.1.2) for Akt consensus phosphorylation motifs.^38, 39^ This search revealed that human PHB contains three Akt consensus phosphorylation sites at Thr108, Ser151 and Thr258, which conforms to the optimal Akt motif RXRXXp(S/T)^38^ (Figure 5d). Therefore, clones/mutants (mutant Thr108Ala, Ser151Ala or Thr258Ala human Flag-tagged PHB) were produced and transfected into T24 cells, followed by treatment with or without the Akt inhibitor GSK690693. Pellets of immunoprecipitated Flag-PHB were probed with PAS antibody. The results showed that PAS antibody detected phosphorylated Flag-PHB-Thr108Ala and Flag-PHB-Ser151Ala in Flag-PHB immunoprecipitates, and GSK690693 inhibited the phosphorylation of these Flag-PHB (Figure 5e). In contrast, PAS antibody failed to detect the phosphorylation of Flag-PHB-Thr258Ala (Figure 5e), indicating Thr258 as an authentic Akt phosphorylation site.

### Akt directly regulates the phosphorylation of PHB

**Figure 6:**
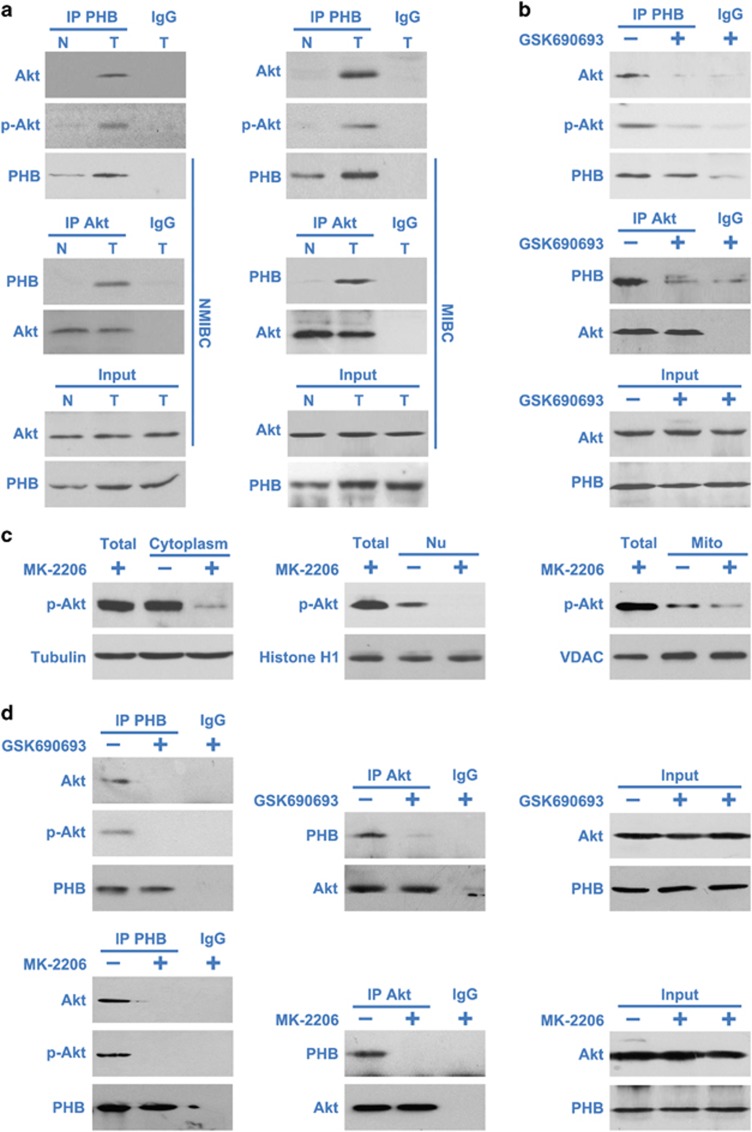
Akt directly regulates the phosphorylation of PHB. (**a**) NMIBC/MIBC and matched tumor-adjacent morphologically normal bladder epithelial tissue lysates were immunoprecipitated (IP) using PHB antibody and analyzed by western blotting (WB) using the indicated antibodies (left panel). NMIBC/MIBC and matched tumor-adjacent morphologically normal bladder epithelial tissue lysates were immunoprecipitated with Akt antibody, and PHB, Akt and activated phospho-Akt levels were analyzed by WB (middle panel). Equal amounts of total input PHB and Akt (Input) were used for immunoprecipitations for each condition (right). (**b**) T24 cells were treated with or without Akt inhibitor GSK690693 (5 *μ*M) for 12 h. Then cell lysates were immunoprecipitated using PHB antibody (upper panel) or Akt antibody (middle panel) and analyzed by WB using the indicated antibodies. Equal amounts of total input PHB and Akt (Input) were used for immunoprecipitations for each condition (lower panel). (**c**) T24 cells were treated with or without Akt inhibitor MK-2206 (5 *μ*M) for 12 h. Cell cytoplasm, nuclei (Nu) and mitochondria (Mito) were separated and analyzed by WB using p-Akt, Histone H1 (nucleus marker), VDAC (mitochondrial marker) and Tubulin (cytoplasm marker) antibodies; Total, total tissue lysates. (**d**) T24 cells were treated with or without Akt inhibitor GSK690693 (5 *μ*M) or MK-2206 (5 *μ*M) for 12 h. Cell cytoplasm were separated for immunoprecipitation using PHB antibody or Akt antibody and analyzed by WB using the indicated antibodies. N, adjacent normal bladder epithelial; T, BC tissue. The results are representative of three independent experiments

To explore the possibility that Akt may directly phosphorylate PHB, we analyzed the immunoprecipitated pellet of endogenous PHB for the presence of Akt. In NMIBC or MIBC tissues, immunoblot analysis of immunoprecipitated PHB detected the presence of Akt and activated phospho-Akt (Figure 6a). We also found PHB present following a reciprocal immunoprecipitate using the antibody against PHB (Figure 6a), further confirming their direct interaction.

To confirm that endogenous Akt interaction with PHB is dependent on Akt activity, T24 cells were treated with or without GSK690693, lysed and immunoprecipitated using the antibody against PHB or Akt. Immunoblot analysis affirmed the interaction of PHB and activated phospho-Akt, and the inhibition of Akt completely disrupted the interaction between PHB, Akt and activated phospho-Akt (Figure 6b). These data indicate that Akt interacts with PHB in BC cells and the activation of Akt is required for the interaction of PHB and Akt.

It was interesting to examine whether the two proteins interacted with each other in different compartments of the BC cells. Nuclear, mitochondrial and cytoplasmic (without mitochondrion) extracts were prepared from control T24 cells or T24 cells treated with Akt inhibitor MK-2206 for 12 h. Western blotting showed that active phospho-Akt was present in the cell nuclear, mitochondrial and cytoplasmic extracts (without mitochondrion) (Figure 6c). But the interaction between PHB and Akt were only detected in the cytoplasmic (without mitochondrion) extract of control cells (Figure 6d) but not in the nuclear or mitochondrial extracts (data not shown). These experiments confirm that PHB is being phosphorylated by Akt in the cytoplasm.

### Phosphorylation-mediated mitochondrial localization of PHB

**Figure 7:**
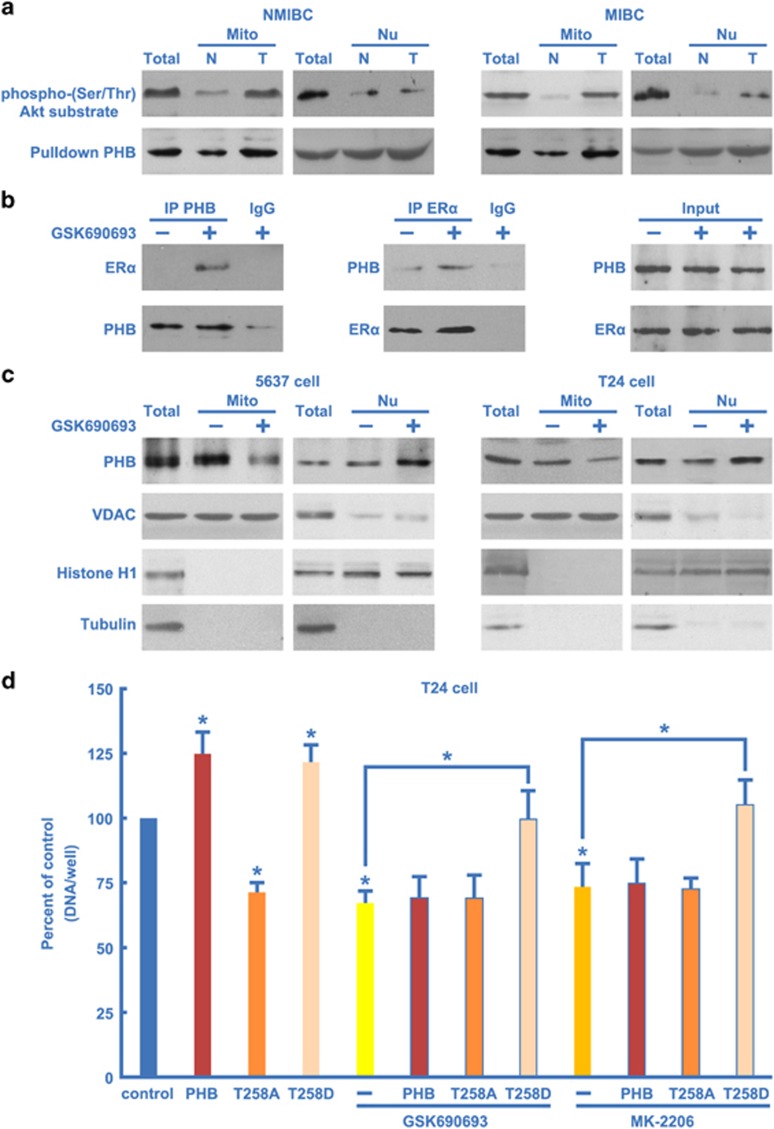
Phosphorylation-mediated mitochondrial localization of PHB is required for enhanced cell proliferation. (**a**)NMIBC or MIBC and matched tumor-adjacent morphologically normal bladder epithelial tissue mitochondria (Mito) and nuclei (Nu) were separated and analyzed by western blotting (WB) using anti-PHB or anti-Phospho-(Ser/Thr) Akt substrate (PAS) antibody; T, total tissue lysates. (**b**) 5637 or T24 cells were treated with or without Akt inhibitor GSK690693 (5 *μ*M) for 12 h. Then cell lysates were immunoprecipitated (IP) using PHB antibody (left) or ER*α* antibody (middle) and analyzed by WB using the indicated antibodies. Equal amounts of total input PHB and ER*α* (Input) were used for IPs for each condition (right). (**c**) 5637 or T24 cells were treated with or without Akt inhibitor GSK690693 (5 *μ*M) for 12 h. Cell mitochondria (Mito) and nuclei (Nu) were separated and analyzed by WB using PHB, Histone H1 (nucleus marker), VDAC (mitochondrial marker) and Tubulin (cytoplasm marker) antibodies; T, total tissue lysates. (**d**) T24 cells were transfected with wild-type Flag-PHB, Flag-PHB T258A or Flag-PHB T258D for 24 h and then were further treated with or without Akt inhibitor GSK690693 (5 *μ*M) or MK-2206 (5 *μ*M) for 12 h, and cell proliferation was quantified. N, adjacent normal bladder epithelial; T, BC tissue. The values represent the mean±S.E. of at least three independent experiments. *Denotes *P*<0.05

Once we had established Akt's critical role in the process, we further investigated the phosphorylation effects on PHB and its localization. Interestingly, in the NMIBC or MIBC tissue, levels of phosphorylated PHB were strikingly upregulated in the mitochondria but not in the nucleus (Figure 7a). These results suggest that the phosphorylated PHB is mainly located in the mitochondria, not in the nucleus.

Previous studies have shown that PHB is mainly localized and functions within the mitochondria,^17^ and we found that mitochondrial PHB translocates to the nucleus in the presence of ER*α* in androgen-independent prostate cancer PC3 cells.^19^ In BC cells, it was reported that ER*α* suppresses BC initiation and invasion, whereas ER*β* promotes BC initiation and progression.^40^ Mechanistic studies suggest that ER*α* and ER*β* exert these effects via modulation of the Akt pathway and DNA replication complex.^41, 42^ Yet, it remains unclear how PHB is delivered to the mitochondria in BC cells.

To further confirm whether Akt-induced PHB phosphorylation could mediate the translocation of PHB, we analyzed the interaction between PHB and ER*α*. In 5637 or T24 cell lines, immunoblot analysis of immunoprecipitated PHB detected neither the presence of ER*α* nor ER*β* (Figure 7b, data not shown). Also, PHB was not present following a reciprocal immunoprecipitate using the antibody against ER*α* (Figure 7b). Furthermore, we found that PHB interacted with ER*α* upon Akt inhibitor treatment (Figure 7b). Furthermore, following Akt inhibitor treatment, PHB levels were elevated in the nuclear fractions and decreased in the mitochondrial fractions as compared with untreated BC cells (Figure 7c). These results indicate that phosphorylation of PHB could disrupt the interaction between ER*α* and PHB and mediate the mitochondrial localization of PHB.

### Phosphorylation of PHB is required for enhanced cell proliferation

Based on the importance of PHB and Akt in BC cell proliferation, we hypothesized that phosphorylation of PHB by Akt is necessary for the proliferation effects of PHB. The overexpression of wild-type PHB, mutant PHB T258A, constitutively activated PHB T258D and Akt inhibitors were used to treat the BC cell lines. Wild-type PHB alone had an obvious effect on proliferation, whereas the mutant PHB T258A and the Akt inhibitor had a remarkable effect on inhibition of cell proliferation (Figure 7d). In contrast, constitutively activated PHB T258D significantly induced profileration and even removed the barrier of proliferation inhibition conferred by the Akt inhibitors (Figure 7d). These findings demonstrate that phosphorylation of PHB is required for BC cell proliferation.

## Discussion

Previous work has demonstrated that overexpression of PHB was not found to correlate with pro-tumorigenic function.^27^ Our results validated that PHB is frequently overexpressed in BC tissues and that this is significantly correlated with tumor recurrence and advanced T and N stage, suggesting that upregulation of PHB in BC may facilitate the invasive/metastatic phenotype. In addition, these findings may underscore the potentially important role of PHB as one of the major underlying biological mechanisms of BC development and progression.

The subcellular localization of PHB has been shown to affect cell fate.^19, 43^ Phosphorylation is also a fundamental posttranslational modification that is used to regulate PHB function, localization and binding specificity of the proteins.^25, 44^ PHB has been reported to interact with Akt and to have a modulatory role in the PI3K/Akt pathway. A recent study has shown that overexpressed Akt phosphorylates PHB on Thr258 *in vitro* and that it is also capable of doing so *in vitro* on overexpressed PHB in HEK-293T cancer cells.^25^ Our group is particularly interested in understanding the functional significance of Akt-induced phosphorylation on PHB and its downstream effects on BC cells. Our data suggest the phosphorylation of PHB by Akt promotes cell proliferation, in contrast to much evidence suggesting the inhibitory effectors of PHB on cancer. Until now, there is no clear mechanism that explains these two disparate conclusions. In the present study, we found that endogenous Akt directly regulates the phosphorylation Thr258 of PHB. Furthermore, constitutively activated PHB T258D significantly induce proliferation and removed the inhibition conferred by suppressing Akt. We demonstrate here for the first time that PHB through phosphorylate at Thr258 to acquire the proliferation activity in PI3K/Akt-dependent pathway.

Interestingly, previous researchers have only observed the phosphorylation of exogenously expressed PHB in HEK-293T cells and only in the presence of exogenously expressed Akt.^25^ Our study shows that in both human BC tissues and cultured human BC cells Thr258 of endogenous PHB is phosphorylated. We also found that PHB expression is upregulated and associated with the high activation of Akt in BC tissues. These results indicate that both the upregulation of PHB and the heightened activation of Akt are required for the phosphorylation of Thr258 on PHB and further support the association of PHB upregulation with poor prognosis in BC patients.

In the nucleus, PHB was found to interact with the transcriptional regulators retinoblastoma-associated protein and E2F and to inhibit transcription from E2F-responsive promoters and thereby suppress cell proliferation.^10, 11, 18, 19^ On the contrary, mitochondrial PHB has been proposed to have an important role in tumorigenesis.^14, 16, 17^ Our group is very interested in the mechanism of PHB function conversion, and our previous studies have shown that, in androgen-independent prostate cancer cells, PHB could physically interact with ER*α*, and ER*α* directly mediates the PHB mitochondrial–nuclear translocation, thus inhibiting cell proliferation.^19^ In the current work, we found that phosphorylation of PHB at Thr258 could disrupt the interaction between ER*α* and PHB and mediate the mitochondrial localization of PHB. Further studies are needed to elucidate the partner or mediator molecules that guide PHB into mitochondria during the progression from normal to cancerous. Targeting specific subsets of cellular PHB may have therapeutic potential, and reducing mitochondrial PHB and/or increasing nuclear PHB may inhibit tumor progression.

PHB and PHB2 (also called prohibitin 2) are two closely related proteins that always form a multimolecular complex in the inner membrane of mitochondria^45^ to regulate cell survival,^29, 46^ proliferation^14^ and energy metabolism.^14^ But, in the nucleus, previous studies show that PHB and PHB2 always interact with a variety of different nuclear proteins involved in gene transcription.^47^ Interestingly, it has been reported that Akt-mediated phosphorylation of PHB2 at Ser91 is required for mouse embryonic fibroblast cell apoptotic resistance,^48^ whereas phosphorylation of PHB2 at the same site by active CaMK IV de-represses the transcriptional inhibitory effect of PHB2 on myocyte enhancer factor-2 activity.^49^ These evidences indicate that Akt and CaMK IV may not only act as protein kinase to induce the phosphorylation of PHBs but may also act as scaffold to recruit and activate the different partner or mediator molecules of PHBs.

In conclusion, this study has identified PHB as a *bona fide* target of Akt kinase for the first time. Our findings demonstrate that Akt phosphorylates PHB at Thr258 and promotes PHB mitochondrial translocation to induce BC proliferation and highlights PHB as an important regulator during BC tumorigenesis.

## Materials and Methods

### Cell culture and treatment

5637, BIU87, T24 and EJ cells were obtained from the American Type Culture Collection (Manassas, VA, USA) and cultured at 37 °C in 5% CO_2_ in DMEM-F12 (1:1) media (Invitrogen Life Technologies, Carlsbad, CA, USA) supplemented with 1% penicillin (100 U/ml, Invitrogen Life Technologies), 1% streptomycin (100 *μ*g/ml, Invitrogen Life Technologies), L-glutamine (292 *μ*g/ml, Invitrogen Life Technologies) and 10% fetal bovine serum (HyClone Laboratories, Logan, UT, USA). GSK690693 (Sigma-Aldrich, St. Louis, MO, USA; 5 *μ*M) or MK-2206 (Selleckchem, Houston, TX, USA; 5 *μ*M) was added to the media at the indicated times. For transient gene transfection of cells, Lipofectamine 2000 (Invitrogen Life Technologies) was used.

### Patient information and tissue specimens

BC and adjacent normal tissue samples were obtained with informed consent under institutional review board-approved protocols. The samples were collected between January 2000 and 30 December 2008 at Sun Yat-Sen University Cancer Center (Guangzhou, China). The BC cases selected were based on a clear pathological diagnosis after surgery, follow-up data and had not received neoadjuvant chemotherapy or radiotherapy. Tumour stage was defined according to the 2004 American Joint Committee on Cancer/International Union Against Cancer tumor–node–metastasis classification system. This study was approved by the institute research ethics committee of Sun Yat-Sen University Cancer Center.

This study included 241 patients who had received surgery with transitional-cell carcinoma of bladder. Of these patients, 161 patients underwent radical cystectomy (RC) and 80 patients underwent transurethral resection of bladder tumor (TURBT) at our center. After TURBT, 50 mg THP or Mitomycin C was used in intravesical therapy as intravesical injection immediately or within 24 h. The 241 paraffin-embedded BC samples were analyzed by immunohistochemistry (IHC) to determine the relationship between PHB expression and clinico-pathological features and prognosis. Tumor–node–metastasis staging was determined according to the 2004 American Joint Committee on Cancer tumor–node–metastasis classification of BC. Tumors were graded according to the World Health Organization 2004 guidelines.

### Paired tumor and tumor-adjacent tissues

Twenty pairs of BC tissues and matched tumor-adjacent morphologically normal bladder epithelial tissues were frozen and stored in liquid nitrogen until used to compare the expression levels of PHB protein and mRNA.

### Quantitative PCR (Q-PCR)

Total RNA was extracted and isolated from cells using the TRIzol reagent (Invitrogen Life Technologies) as described previously. First-strand cDNA was synthesized from 1 *μ*g of mRNA using Superscript III reverse transcriptase (Invitrogen Life Technologies) and oligo (dT) as primers. Q-PCR was performed in triplicate on an ABI Prism 7000 sequence detection system (Applied Biosystems, Foster City, CA, USA) using an ABI SYBR Green PCR mixture (Applied Biosystems) as described by the manufacturer. PCR cycling conditions were as follows: initial denaturation at 95 °C for 5–10 min followed by 40 cycles of 95 °C for 30 s, 1 min of annealing, and 1 min of extension at 72 °C. The annealing temperature was adapted for the specific primer set used. Fluorescence data were collected during the annealing stage of amplification, and specificity of the amplification was verified by melting curve analysis. Cycle threshold (Ct) values were calculated using identical threshold values for all experiments. *β-Actin* was used as a control and for normalization. Relative RNA expression was calculated using the formula ratio=2(Ct_ref_−Ct_target_). Data shown represent the mean and S.E. of three separate experiments. The following primer pairs were used: for *phb*, forward (5′-AAACAGGTGGCTCAGCAGGAA-3′) and reverse (5′-CAGTGAGTTGGCAATCAGCTCAG-3′); and for *β-actin*, forward (5′-CGTCTTCCCCTCCATCG-3′) and reverse (5′-CTCGTTAATGTCACGCAC-3′).

### Immunoblotting

Western blotting was performed as described previously.^19, 50^ After determining the protein content of the cell lysates, the protein extracts were separated by 10% SDS-PAGE, transferred to a PVDF membrane and incubated with primary antibody (ER*α*, Akt, p-Akt Ser473, p-GSK-3*β* Ser9, PHB and VDAC antibodies were from Santa Cruz Biotechnology, Santa Cruz, CA, USA, and Tubulin and Histone H1 antibodies were from Abcam, Cambridge, MA, USA). The signal was detected by ECL detection system (GE Healthcare, Buckinghamshire, UK).

### IHC analysis

The 241 tissue blocks were cut into 5-*μ*m sections and processed for IHC using a standard two-step technique as demonstrated previously.^50^ PHB expression was determined by IHC using a rabbit anti-PHB antibody (1 : 200; Santa Cruz Biotechnology), and normal goat serum was used as a negative control. The degrees of immunostaining of formalin-fixed paraffin-embedded sections were viewed and scored by two independent pathologists who were blinded to the histopathological features and patient data of the samples. Both positive areas and staining intensity were recorded. A staining index (values 0–9) obtained as the intensity of PHB-positive staining (weak, 1; moderate, 2; strong, 3) and the proportion of immunopositive cells of interest (0–10%, 0; 11–50, 1; 51–80, 2; >80%, 3) were calculated. Finally, the cases were classified into two different groups: negative expression cases (score 0–3) and cases with positive expression (scores 4–9).

All statistical analyses were performed by using the SPSS 16.0 statistical software package (SPSS-China, Guangzhou, China). We plotted survival curves by the Kaplan–Meier method and compared using the log-rank test for survival analysis. Survival data were evaluated using univariate and multivariate Cox regression analyses. The association with survival and each variable were determined with the log-rank test. Multiple Cox-proportional hazards regression was carried out to identify the protein marker as an independent predictor of survival. Differences was considered significant if the *P*- value from a two-tailed test was <0.05.

### DNA growth assay

Following treatment of cells, the media was discarded, cells were solubilized for 30 min at 37 °C in 0.1% SDS and the amount of DNA was estimated using a Hoechst 33258 microassay, as extensively described previously.^19^

### Subcellular fractionation

Approximately 10^7^ cells were harvested into 10 ml of isotonic fractionation buffer (250 mM sucrose, 0.5 mM EDTA, 20 mM Hepes and 500 *μ*M Na_3_VO_4_ at pH 7.2) supplemented with protease inhibitor cocktail complete (Roche Molecular Biochemicals, Mannheim, Germany) and centrifuged at 900 × *g* for 5 min. The pellet was then resuspended in 200 *μ*l fractionation buffer, homogenized with a ball-bearing homogenizer and centrifuged at 900 × *g* for 5 min to remove the nuclei. The postnuclear supernatant was centrifuged at 20 000 × *g* for 15 min to collect the heavy membrane fraction enriched in mitochondria; the supernatant was as cytoplasm without mitochondrion.

### Co-immunoprecipitation

Cell extracts were prepared by solubilizing 10^7^ cells in 1 ml of cell lysis buffer made of 1% Triton X-100, 150 mM NaCl, 20 mM Tris-Cl at pH 7.4, 1 mM EDTA, 1 mM EGTA, 1 mM Na3VO4, 2.5 mM pyrophosphate, 1 mM glycerol phosphate and protease inhibitor mixture for 10 min at 4 °C. After brief sonication, the lysates were cleared by centrifugation at 15 000 × *g* for 10 min at 4 °C, the cell extract was immunoprecipitated with 6 *μ*g of antibodies against ER*α*, Akt or PHB (antibodies were from Santa Cruz Biotech) and incubated with 100 *μ*l of protein G plus protein A-agarose for 12 h at 4 °C by continuous inversion. Immunocomplexes were pelleted, washed four times, boiled in Laemmli buffer and analyzed by western blotting.

### Cell survival assays

Cells were stained with Hoechst 33258 (5 *μ*g/ml) to visualize nuclear morphology. Apoptosis was quantified by scoring the number of cells with pyknotic nuclei (chromatin condensation or nuclear fragmentation) relative to the total number of Hoechst 33258-positive cells in the same visual field. Cells were counted in an unbiased manner (at least 1000 cells for each group) and were scored blindly without previous knowledge of their treatment.

### Constructs

The plasmids pCDNA3.1-3xFlag-PHB was made by inserting Human Prohibitin cDNA into a pCDNA3.1-3xFlag expression vector. The threonine or serine substitutions to alanine (A) or Aspartate (D) in the flag-PHB T108A, Ser151A, Thr258A and Thr258D were generated by site-directed mutagenesis (QuikChange Site-Directed Mutagenesis Kit, Stratagene, La Jolla, CA, USA). Constructs were transfected into cells using Lipofectamine 2000.

### Statistical analysis

All statistical analyses were performed using PASW Statistics 18.0 (SPSS Inc., Chicago, IL, USA), with the exception of the significance in bar graphs, in which case analyses were performed by applying the independent *t*-test using the Microsoft Office Excel software (Microsoft Corp., Redmond, WA, USA). The statistics in the graphs represent the means with±S.E. bars of at least three independent experiments. A *P*-value of <0.05 was considered significant.

## Figures and Tables

**Table 2:** Univariate and multivariate analysis of different prognostic parameters associated with overall survival of 161 patients with MIBC

**Variable**	**Univariate analysis^a^**		**Multivariate analysis^b^**	
	**All cases**	***P*-value**	**HR (95% CI)**	***P*-value**
*Gender*		0.226		
Male	16			
Female	145			
				
*Age (years)*		0.115		
<60	77			
≥60	84			
				
*Tumor multiplicity*		0.580		
Unifocal	64			
Multifocal	97			
				
*Tumor size (cm)*		0.748		
<5	88			
≥5	73			
				
*pT status*		0.007		
pT1	32		1	
pT2	80		1.064 (0.332–1.755)	0.024
pT3	30		2.180 (0.929–5.113)	0.013
pT4	19		4.935 (2.048–11.892)	0.000
				
*pN status*		0.036		0.021
N−	143		1	
N+	18		2.238 (1.124–4.448)	
				
*Histological grade*		0.015		0.250
Low	43		1	
High	118		1.715 (0.404–2.266)	
				
*PHB^+^ expression*		0.019		0.045
Negative	50		1	
Positive	111		1.176 (0.700–1.973)	

a Univariate analysis, log-rank test.

b Multivariate analysis, Cox regression model

**Table 1:** Correlation between PHB expression and clinicopathological features in 241 cases of BC

**Characteristics**	**All cases** **(*n*, NMIBC/MIBC)**	**PHB expression (NMIBC/MIBC)**		
		**Negative no. (%)**	**Positive no. (%)**	*P*-value*
*Gender*				0.332/0.226
Male	18/16	5 (27.8%)/3 (18.8%)	13 (72.2%)/13 (81.2%)	
Female	62/145	23 (37.1%)/47 (32.4%)	39 (62.9%)/98 (67.6%)	
				
*Age (years)*				0.254/0.379
<60	51/77	16 (31.4%)/26 (33.8%)	35 (68.8%)/51 (66.2%)	
≥60	29/84	12 (41.4%)/24 (28.6%)	17 (58.6%)/60 (72.4%)	
				
*pT status*				None/0.009
pT1	80/32	28 (35%)/12 (37.5%)	52 (65%)/20 (63.5%)	
pT2	0/80	0/19 (23.7%)	0/61 (76.3%)	
pT3	0/30	0/10 (33.3%)	0/20 (66.7%)	
pT4	0/19	0/9 (47.3%)	0/10 (52.6%)	
				
*pN status*				None/0.027
N−	80/143	28 (35%)/43 (30.1%)	52 (65%)/100 (69.9%)	
N+	0/18	0/7 (38.9%)	0/11 (61.1%)	
				
*Histological grade*				0.022/0.259
Low	49/43	15 (30.6%)/16 (37.2%)	34 (69.4%)/27 (62.8%)	
High	31/118	13 (41.9%)/34 (28.8%)	18 (58.1%)/84 (71.2%)	
				
*Tumor multiplicity*				0.016/0.048
Unifocal	66/64	21 (31.8%)/23 (35.9%)	45 (68.2%)/41 (64.1%)	
Multifocal	14/97	7 (50%)/27 (27.8%)	7 (50%)/70 (72.1%)	
				
*Tumor size (cm)*				0.376/0.401
<5	55/88	21 (38.2%)/25 (28.4%)	34 (68.1%)/63 (71.6%)	
≥5	25/73	7 (28.0%)/25 (34.2%)	18 (72.0%)/48 (65.8%)	
				
*UIS*				0.350/0.898
Yes	14/24	6 (42.9%)/11 (45.8%)	8 (57.1%)/13 (54.2%)	
No	66/137	22 (33.3%)/39 (28.5%)	44 (66.7%)/98 (71.5%)	

Abbreviation: UIS, urinary irritation symptoms. *P-value, *X*^2^ test

## Abbreviations

BC: bladder cancer
Akt: RAC-alpha serine/threonine-protein kinase
PHB: Prohibitin 1
PI3K: phosphatidylinositol-4-phosphate 3-kinase
